# Clinical care for sexual assault survivors multimedia training: a mixed-methods study of effect on healthcare providers’ attitudes, knowledge, confidence, and practice in humanitarian settings

**DOI:** 10.1186/1752-1505-7-14

**Published:** 2013-07-03

**Authors:** Janel R Smith, Lara S Ho, Anne Langston, Neha Mankani, Anjuli Shivshanker, Dhammika Perera

**Affiliations:** 1 International Rescue Committee, 122 East 42nd Street, New York, NY 10168, USA; 2 Department of International Health, Johns Hopkins Bloomberg School of Public Health, 615 North Wolfe Street, Baltimore, MD 21205, USA; 3 Columbia University Mailman School of Public Health, 722 West 168th Street, New York, NY 10032, USA

**Keywords:** Sexual and Gender Based Violence (SGBV), Sexual assault, Healthcare provider, Training, Humanitarian, Refugee, Democratic Republic of Congo (DRC), Ethiopia, Kenya, Jordan

## Abstract

**Background:**

Sexual assault is a threat to public health in refugee and conflict affected settings, placing survivors at risk for unintended pregnancy, unsafe abortion, STIs, HIV, psychological trauma, and social stigma. In response, the International Rescue Committee developed a multimedia training tool to encourage competent, compassionate, and confidential clinical care for sexual assault survivors in low-resource settings. This study evaluated the effect of the training on healthcare providers’ attitudes, knowledge, confidence, and practices in four countries.

**Methods:**

Using a mixed-methods approach, we surveyed a purposive sample of 106 healthcare providers before and 3 months after training to measure attitudes, knowledge, and confidence. In-depth interviews with 40 providers elaborated on survey findings. Medical record audits were conducted in 35 health facilities before and 3 months after the intervention to measure healthcare providers’ practice. Quantitative and qualitative data underwent statistical and thematic analysis.

**Results:**

While negative attitudes, including blaming and disbelieving women who report sexual assault, did not significantly decrease among healthcare providers after training, respect for patient rights to self-determination and non-discrimination increased from 76% to 91% (p < .01) and 74% to 81% (p < .05) respectively. Healthcare providers’ knowledge and confidence in clinical care for sexual assault survivors increased from 49% to 62% (p < .001) and 58% to 73% (p < .001) respectively following training. Provider practice improved following training as demonstrated by a documented increase in eligible survivors receiving emergency contraception from 50% to 82% (p < .01), HIV post-exposure prophylaxis from 42% to 92% (p < .001), and STI prophylaxis and treatment from 45% to 96% (p < .01).

**Conclusions:**

Although beliefs about sexual assault are hard to change, training can improve healthcare providers’ respect for patient rights and knowledge and confidence in direct patient care, resulting in more competent and compassionate clinical care for sexual assault survivors.

## Background

Sexual assault is a global public health and human rights challenge, and a particular threat to refugee, internally displaced, and post-conflict populations [1-3]. At least one in three of the world’s female population is either physically or sexually abused in her lifetime and one in four women experience sexual violence by an intimate partner [1]. Sexual assault increases during times of conflict when rape is used as a weapon of war or perpetrated within a climate of impunity [2,3].

The *Minimum Initial Service Package (MISP) for Reproductive Health in Crisis Situations* includes clinical management for survivors of sexual assault and is part of the Sphere Project’s *Minimum Standards in Humanitarian Response*[4]. The consequences of sexual assault on a survivor’s physical and mental health are well documented and may include physical injury, sexually transmitted infections (STIs) including HIV, unwanted pregnancy, unsafe abortion, anxiety, shame, post-traumatic stress, and depression [3]. Timely access to clinical care, delivered by competent and compassionate healthcare providers (HCPs), is essential to prevent adverse consequences and begin a survivor’s physical and emotional healing.

However, limited clinical competency and negative attitudes among HCPs inhibit care seeking, lead to poor quality services, and contribute to survivors’ re-traumatization [5-10]. The fear of stigma and associated marginalization is substantial and has a significant impact upon survivors’ reporting of sexual violence. In a study of sexual assault in post-conflict settings within DRC, only 46% sought healthcare within 72 hours after sexual assault [5]. A study in Uganda suggested that as much as 90% of sexual assault which occurred in IDP camps remained unreported [6]. A global review of health care-based interventions for survivors of sexual violence revealed that a lack of clinical competency and negative attitudes are prevalent among healthcare providers and often result in poor quality health services [7]. A study in South Africa showed that only 30% of HCPs had received training on the clinical management of rape and only 32% considered rape to be a serious medical problem [8]. Further studies show that use of poor quality health services can be a negative and disempowering experience for rape survivors [9,10].

Training HCPs is associated with positive effects on the quality of clinical care delivery and health and psychosocial outcomes for survivors of sexual assault [11-15]. In the United States, evaluations of the sexual assault nurse examiner (SANE) training program showed improvements in post-rape medical care and better psychological recovery for survivors [11-13]. A study in Latin American countries found that negative attitudes were prevalent among HCP but improved following training [14]. In South Africa, a clinical care training for providers resulted in improvements in clinical history and examination, pregnancy testing, emergency contraception, prophylaxis for sexually transmitted infections; HIV counseling and testing, PEP, trauma counseling, and referrals [15].

To date few studies have evaluated the effectiveness of HCP training for improved clinical care of sexual assault in humanitarian settings and there is increasing demand to build an evidence base around training tools and methods effective in humanitarian settings [16]. In response, the International Rescue Committee (IRC) implemented and evaluated the effectiveness of the Clinical Care for Sexual Assault Survivors (CCSAS) multimedia training tool in four humanitarian settings [17]. The tool has been endorsed by the Reproductive Health Response in Crisis (RHRC) Consortium and used by IRC in partnership with UNHCR, ministries of health, and various non-governmental organizations in humanitarian settings globally to encourage competent, compassionate, and confidential clinical care for sexual assault survivors.

### Objective

This paper describes the effect of the IRC CCSAS multimedia training on the attitudes, knowledge, confidence, and practices of HCPs providing clinical care to sexual assault survivors in refugee camps in Ethiopia and Kenya, a post-conflict setting in the Democratic Republic of Congo (DRC), and an urban refugee setting in Jordan.

### Intervention

The training program utilized the IRC CCSAS multimedia training tool designed to improve the clinical care of sexual assault survivors in diverse low resource settings. The tool includes video re-enactment of interactions between HCPs and survivors of sexual assault to model best practice, interviews with clinical care experts from around the world, case studies, group exercises, role plays, and participant hand-outs. The training tool covers content on:

• What every clinic worker needs to know including the global burden of sexual assault, beliefs affecting sexual assault survivors, and survivors’ patient rights.

• Responsibilities of non-medical staff including receptionists, interpreters, cleaning staff, and security personnel.

• Direct patient care including receiving a sexual assault survivor and conducting a preliminary assessment; obtaining informed consent and taking the patient’s history; performing a physical exam; providing treatment and disease prevention; caring for male survivors; and caring for child survivors.

• Preparing your clinic including addressing the clinic’s resources, organizing staff and materials needed to care for survivors, and mapping the referral network.

Trainings were co-facilitated by health and gender based violence specialists using the multimedia training tool according to the methodology presented in the facilitator’s guide [17]. Trainings took place over 4 days and included all sections described above. All trainings were provided in the working language specific to the site.

## Methods

### Study settings

The study was conducted from November 2010 to June 2012 in multiple humanitarian settings across four countries and diverse geographical regions. It was assumed that cultural and contextual factors would influence HCPs’ knowledge, attitudes, and practices in the clinical care of sexual assault survivors, and subsequently, their uptake of the training. Therefore, study settings were purposively selected to demonstrate the effectiveness of the CCSAS Multimedia Training Tool in a diversity of humanitarian settings, health facility levels, and geographical regions. Study settings included an IRC operated hospital in Dadaab Refugee Camp, Kenya, two government operated hospitals in Sheder and My’Ayni Refugee Camps in Ethiopia, two health facilities operated by a local non-governmental organization in an urban refugee setting in Amman, Jordan, and two referral hospitals and twenty-eight health posts operated by the government in post-conflict settings in Kabare and Kalehe health zones, South Kivu, Democratic Republic of Congo. All study sites, except Jordan, demonstrated previous documented cases of sexual violence in health facilities and all study sites had no previous training for health facility staff on clinical care for sexual assault, although some had received related training on gender based violence.

### Study design

A mixed-methods study evaluated changes in HCP knowledge, confidence, attitudes, and practices pre and three months post CCSAS training. Data was collected through clinician questionnaires and in-depth interviews with HCPs and medical record audits. Tools were developed based on the content of the training and modelled after existing tools measuring HCP’s knowledge, attitudes, and practices in the clinical care of sexual assault [18,19]. Tools were adapted to the local context and translated into the local language.

• Clinician questionnaires were self-administered by 106 HCPs pre and three months post training. The questionnaire included multiple choice, true/false, and 5-point Likert scales to measure items on demographic information, knowledge of direct patient care protocols and procedures, confidence in providing direct patient care, attitudes toward sexual assault, and practices providing care to survivors.

• In-depth interviews were conducted with 40 HCPs pre and three months post training. The in-depth interview field guide was designed to qualitatively explore HCPs’ knowledge, confidence, attitudes, and practices caring for survivors of sexual assault. Interviews were conducted in English by the PI with interpretation in Amharic, Arabic, French, Somali, or Tigrinya as necessary by multilingual research staff trained in interpretation. Some interviews were conducted in French by a bilingual co-investigator without the use of interpreters. Interviews were audio recorded, transcribed verbatim, and translated into English.

• Medical record audits were conducted retrospectively on all sexual assault cases documented in 35 health facilities in the three months prior to the training intervention and three months post training intervention. Items measured the quality of care delivered to sexual assault survivors according to WHO guidelines and covered essential aspects of care including documentation, consent, incident history, medical history, medical exam, investigations, treatment, and referral [20].

### Study participants

A purposive sample of 106 HCPs were selected to participate in the CCSAS training and survey evaluation. All selected participants had no previous exposure to IRC’s CCSAS training. Among the 106, 40 were purposively selected to participate in in-depth interviews as key informants. Key informants were purposively selected to represent a variety of health care workers, all of whom were not previously trained in clinical care for sexual assault.

### Data analysis

• Quantitative data were analyzed for measures of central tendency. Paired t-tests and chi-squared tests were run on scores to determine statistical significance of changes in scores from baseline prior to training to end-line three months after training. An Analysis of Variance (ANOVA) test was run to determine differences in uptake of the training by country, sex, job title, and previous experience.

• Audio recordings of qualitative data collected through in-depth interviews were transcribed verbatim in the original language (English, Arabic, Amharic, French, Somali, and Tigrinya) by multilingual research staff trained in transcription. Transcripts in Arabic, Amharic, French, Somali, and Tigrinya were then translated and transcribed into English by multilingual research staff trained in transcription. English transcripts were entered into ATLAS.ti and coded for recurrent patterns and themes by the PI and two co-investigators. Coding was guided by a code book developed by the PI which included 35 hierarchical codes related to the training content and including: health care provider attitudes toward sexual assault survivors and respect for patient rights; and health care provider knowledge, confidence, and practice in all aspects of clinical care including assessment, history taking and informed consent, physical exam, treatment, care for male survivors, and care for child survivors. Coded qualitative data were triangulated against quantitative data.

### Ethical issues

Ethical review and approval of the research protocol was obtained from an IRC review board, the Administration for Refugee and Returnee Affairs (ARRA) in Ethiopia, and Kenyatta National Hospital IRB in Kenya. Permission to conduct the evaluation was granted by operating agencies in each site including the MoPH in South Kivu, DRC; Administration for Refugee and Returnee Affairs (ARRA) in My’Ayni and Sheder refugee camps, Ethiopia; the IRC in Hagadera refugee camp, Kenya; and the Jordan Health Aid Society (JHAS) in Amman, Jordan. Written or verbal consent was obtained from respondents prior to conducting surveys or interviews. The consent form was translated into the local language and included information on the purpose of the study, the risks and benefits of participating in the study, the voluntary nature of the study, and procedures for protecting the confidentiality of information obtained. All data was collected anonymously using an identification code and no personal identifiers were recorded.

## Results

### Demographics

A total of 106 HCPs from DRC, Ethiopia, Kenya, and Jordan participated in the CCSAS training and completed the clinician questionnaire (Table 1). The majority of HCPs surveyed were male (65.1%) and under the age of 30 (57.5%). Nurses and midwives made up a larger proportion of participants (70.7%) as compared to doctors (10.4%), and auxiliary technicians and nurse aides (18.9%). Overall, half of participants reported some previous experience providing direct patient care to sexual assault survivors. HCPs working in a refugee camp in Kenya had the most experience (70.4%), while those working in an urban refugee setting in Jordan had the least experience (21.4%).

**Table 1:** Healthcare provider demographics

	**DRC**	**Ethiopia**	**Kenya**	**Jordan**	**Total**
	**n (%)**	**n (%)**	**n (%)**	**n (%)**	**n (%)**
Total	39	26	27	14	106
Sex					
Female	10(25.6%)	10(38.5%)	6(22.2%)	9(64.3%)	35(33.0%)
Male	29(74.4%)	16(61.5%)	19(70.4%)	5(35.7%)	69(65.1%)
NR	0(0.0%)	0(0.0%)	2(7.4%)	0(0.0%)	2(1.9%)
Age					
<30	15(38.5%)	19(73.1%)	19(70.4%)	8(57.1%)	61(57.5%)
30-39	14(35.9%)	5(19.2%)	4(14.8%)	3(21.4%)	26(24.5%)
>39	10(25.7%)	2(7.7%)	2(7.4%)	3(21.4%)	17(16.0%)
NR	0(0.0%)	0(0.0%)	2(7.4%)	0(0.0%)	2(1.9%)
Job title					
Doctor	2(5.0%)	1(3.8%)	3(11.1%)	5(35.7%)	11(10.4%)
Nurse/Midwife	37(95.0%)	20(76.9%)	15(55.5%)	3(21.4%)	75(70.7%)
Auxiliary	0(0.0%)	5(19.2%)	9(33.3%)	6(42.9%)	20(18.9%)
Previous Experience					
Yes	20(51.3%)	11(42.3%)	19(70.4%)	3(21.4%)	53(50%)
No	19(48.7%)	15(57.7%)	8(29.6%)	11(78.6%)	53(50%)

### Attitudes

Summary scores based on agreement with positive statements and disagreement with negative statements revealed an overall average improvement in HCP attitudes from 72% to 77% (p < .01) (Table 2). Groups of HCPs demonstrating more positive attitudes included females and those with experience caring for survivors. Female HCPs’ attitudes improved from 77% to 85% (p < .01), while male HCPs’ attitudes averaged 70% before training and did not improve significantly. Attitudes of HCPs with no previous experience caring for survivors improved from 66% to 74% (p < .01), while attitudes of experienced HCPs averaged 74% before training but did not improve significantly.

**Table 2:** Effect of training on attitudes of HCPs by group

	**n**	**Baseline**	**Endline**	**Difference**	**p -value**
		**Mean (95% CI)**	**Mean (95% CI)**	**Mean (95% CI)**	
Total	106	71.76(66.79-73.14)	77.20(72.53-78.34)	5.44(1.89-8.98)	.003
Country					
DRC	39	71.11(64.73-74.35)	71.94(67.24-75.65)	0.83(−5.27-6.94)	.780
Ethiopia	26	69.69(56.06-72.26)	81.01(69.84-82.65)	11.31(5.78-16.83)	.001
Kenya	27	73.98(68.20-79.81)	84.05(79.26-89.15)	10.06(3.6-16.53)	.005
Jordan	14	71.85(59.27-84.42)	71.85(60.07-81.25)	0.00(−14.22-14.22)	1.00
Sex					
Female	35	77.03(66.25-81.72)	85.33(74.38-85.25)	8.29(2.29-14.29)	.010
Male	69	70.10(65.47-72.63)	74.49(70.00-76.92)	4.39(−0.11-8.89)	.056
Job Title					
Doctor	11	76.88(68.24-85.53)	80.22(67.76-89.80)	3.33(−5.18-11.85)	.399
Nurse/Midwife	75	71.46(65.76-73.52)	76.02(71.49-78.52)	4.56(0.212-8.91)	.040
Auxiliary	20	67.77(57.51-73.59)	78.66(68.31-80.58)	10.88(−0.41-22.19)	.050
Previous Experience					
Yes	53	74.67(68.97-77.17)	78.77(74.25-82.85)	4.09(−0.79-8.91)	.094
No	53	66.22(60.57-70.15)	74.22(68.06-75.17)	8.00(2.95-13.04)	.004

Note: Values are summary scores of HCPs indicating attitudes on a Likert scale on clinician questionnaire.

Two categories of attitudes were measured, the first being beliefs about sexual assault and the second being respect for patient rights. Negative or potentially harmful beliefs about sexual assault, including disbelieving and blaming persons who report sexual assault, were common among HCPs and did not decrease significantly after training. However, respect for patient rights, including the right to non-discrimination and self-determination, increased among HCPs after training. Interviewees stated that the training convinced them that their personal beliefs should not affect the quality of care delivered to patients according to respect for patients’ rights:

“Changing is personal, so maybe both the knowledge and the facts, which are in the training, can help a person to change. Personally, they helped me. Now I know my work is not to judge but only to focus on treatment.” –Nurse, Kenya

### Beliefs about sexual assault

More than half of HCPs disbelieved reports of sexual assault. Before training, 68% of HCPs agreed with the statement: “people often make accusations about sexual assault that are not true”, and did not decrease significantly after training. Interviews with HCPs in refugee camp settings revealed that many HCPs believed that women often lie about sexual assault in order to be resettled:

“They don’t come with medical problems they come for referral. They like to be referred. It is spoken or rumored that they will get a chance of getting resettlement.”–Nurse, Ethiopia

More than one third of HCPs did not recognize sexual violence between intimate partners. Before training, 35% of HCPs agreed with the statement: “if a woman’s husband forces her to have sex, it does not count as sexual assault”, and did not decrease significantly after training. In all settings, HCPs questioned the legitimacy of rape within marriage: “Who knows really is it force? It depends on culture. It depends on instruction and education. Here in rural, a wife doesn’t have a right to refuse. I’m also from here and I’ve never heard a wife complain.” –Nurse, DRC

Blaming attitudes toward survivors of sexual assault was common and did not change significantly after training. Before training, 39% of HCPs agreed with the statement: “a survivor may be to blame for sexual assault because of the way she or he dressed or acted”, and did not decrease significantly after training.

### Respect for patient rights

Respect for patients’ right to non-discrimination improved among HCPs following training. The proportion of HCPs who agreed with the statement: “It is not my responsibility to determine whether or not sexual assault has occurred,” increased from 74% to 81% (p < .05). HCPs who initially asked disbelieving and discriminatory questions of persons reporting sexual assault, after training provided care based on the woman’s report:

Before training: “We have the consultation to determine if it is true or if it is false. First I would ask her where she lives, what she was doing…from what place she was where she became a victim… her occupation, if she sexually sells instead of married …to know if she was really sexually assaulted or not, to help to diagnose.” - Nurse, DRC

After training: “It’s not sure that a provider is able to determine the assault…you have to give the urgent treatments if she really says that she has been sexually assaulted. But you cannot confirm that she has been… only legal experts can determine that.” –Nurse, DRC

HCPs’ respect for patients’ right to self-determination improved following training. The proportion of HCPs who agreed with the statement: “A survivor has a right to choose whether or not to receive an exam or treatment,” increased from 76% to 91% (p < .01). HCPs who initially provided care regardless of the patient’s consent, after training provided care based on the woman’s choice:

“Initially, the ones who refuse examination, before we think they are not serious maybe, or maybe they were just not understanding. But now we really have to respect their decisions and we know that the patients have the right, the universal rights to accept or refuse the interventions that you may offer to them.” –Medical Doctor, Kenya

### Knowledge and Confidence

HCP’s knowledge and confidence in providing clinical care to sexual assault survivors improved three months following training. Summary scores based on questions answered correctly in the knowledge portion of the questionnaire revealed an overall average improvement in HCP knowledge from 49% to 62% (p < .001) (Table 3). Female HCPs’ knowledge improved from 47% to 64% (p < .001) while males improved from 50% to 60% (p < .001). Doctors’ knowledge improved from 60% to 72% (p < .05) and nurses improved from 50% to 61% (p < .001). Knowledge of HCPs with previous experience caring for survivors improved from 54% to 63% (p < .001) and those without experience improved from 43% to 59% (p < .001). Summary scores based on HCP self-ranking of confidence from not at all confident to extremely confident revealed an overall average increase in HCP confidence from 58% to 73% (p < .001) (Table 4). Male HCPs’ confidence improved from 58% to 73% (p < .001), while females’ confidence did not improve significantly after training (p = .07). Nurses’ confidence improved from 58% to 71% (p < .001), while doctors’ confidence did not improve significantly after training (p = .07). Confidence among HCPs with previous experience caring for survivors improved from 61% to 77% (p < .001) and those without experience improved from 54% to 67% (p < .05). Improvement in HCP knowledge following training varied across aspects of direct patient care as revealed by the proportion of HCPs giving correct responses to individual questionnaire items and supported by qualitative data.

**Table 3:** Effect of training on knowledge of HCPs by group

	**n**	**Baseline**	**Endline**	**Difference**	**p-value**
		**Mean (95% CI)**	**Mean (95% CI)**	**Mean (95% CI)**	
Total	106	49.09(45.57-51.34)	61.59 (59.04-64.42)	12.50 (10.29-16.24)	.001
Country					
DRC	39	48.51(44.311-52.02)	59.21(55.30-63.45)	10.70(5.92-16.50)	.000
Ethiopia	26	49.84(43.70-55.74)	61.05(53.62-67.45)	11.20(5.29-16.33)	.000
Kenya	27	54.25(46.55-59.35)	65.22(60.60-71.44)	10.97(7.24-18.89)	.001
Jordan	14	39.36(28.44-48.14)	62.24(55.94-68.54)	22.88(15.62-32.27)	.000
Sex					
Female	35	47.30(41.44-52.97)	64.25(59.92-68.77)	16.94(12.06-22.21)	.000
Male	69	50.36(46.03-52.81)	60.08(56.75-63.70)	9.71(7.15-14.45)	.000
Job Title					
Doctor	11	60.24(45.37-74.19)	72.67(65.10-79.33)	12.43(3.52-21.33)	.019
Nurse/midwife	75	49.53(45.81-51.92)	61.24(58.55-64.56)	11.71(9.12-16.25)	.000
Auxiliary	20	41.29(34.53-46.89)	56.79(48.98-64.27)	15.49(8.03-23.79)	.000
Previous Experience					
Yes	53	54.18(49.74-57.18)	63.82(59.65-68.05)	9.63(6.77-14.00)	.000
No	53	43.99(39.37-47.55)	59.36(56.20-63.02)	15.36(11.44-20.86)	.000

Note: Values are summary scores of HCPs correctly answering items on clinician questionnaire.

**Table 4:** Effect of training on confidence of HCPs by group

	**n**	**Baseline**	**Endline**	**Difference**	**p-value**
		**Mean (95% CI)**	**Mean (95% CI)**	**Mean (95% CI)**	
Total	106	58.16 (53.86-63.90)	72.66 (66.21-74.30)	14.50 (8.22-20.77)	.001
Country					
DRC	39	54.86(46.37-63.34)	70.97(60.95-73.91)	16.11(6.38-25.83)	.002
Ethiopia	26	71.06(63.15-78.11)	77.54(68.89-84.51)	6.48(−6.03-18.99)	.290
Kenya	27	55.55(44.47-65.74)	70.23(66.12-78.42)	14.68(2.67-26.68)	.019
Jordan	14	45.13(34.54-74.62)	75.00(36.04-85.25)	29.86(−12.02-71.74)	.126
Sex					
Female	35	59.96(52.005-72.99)	73.18(60.35-77.41)	13.22(−1.35-27.808)	.073
Male	69	58.00(51.96-63.46)	73.25(66.92-76.06)	15.25(8.53-21.96)	.000
Job Title					
Doctor	11	61.57(40.72-79.27)	83.79(59.02-95.14)	22.22(−2.43-46.87)	.071
Nurse/midwife	75	58.33(52.78-64.43)	71.26(65.62-74.39)	12.93(6.027-19.83)	.000
Auxiliary	20	53.12(44.78-73.96)	70.31(53.65-79.67)	17.18(−8.70-43.08)	.161
Previous Experience					
Yes	53	60.98(54.26-67.11)	76.70(70.29-80.68)	15.71(7.82-23.61)	.000
No	53	54.16(48.40-64.88)	66.93(58.16-70.24)	12.76(2.01-23.52)	.022

Note: Values are summary scores of HCPs indicating level of confidence on a Likert scale on clinician questionnaire.

### Obtaining informed consent and taking the history

The proportion of HCPs able to identify the purpose of obtaining informed consent increased from 68% to 86% following training (p < .001) and the proportion able to identify active listening skills increased from 49% to 66% (p < .001). HCPs conveyed improved understanding of using active listening skills to conduct a health history by not interrupting or rushing the patient, acknowledging the patient’s emotions, and validating the patient’s feelings:

“For example, for us doctors who initially maybe were not emphasizing more on the mental health or the psychological trauma, now we are. Now even, when they are really emotionally affected, you allow them to cry, you allow them…maybe to understand their situation, and you empathize with them generally. You just reassure them.” –Medical Doctor, Kenya

### Performing a physical exam

Following training, 64% of HCPs identified the purpose of the general physical exam, increased from 43% prior to training (p < .001) and the proportion of HCPs who could identify the indications for performing a speculum exam on a female survivor increased from 51% to 68% (p < .001). HCPs who could identify how to give a survivor control over the exam increased from 62% to 82% (p < .001). After training, HCPs described the key aspects of giving a survivor control over the exam including: explaining exam procedures and findings, encouraging the patient to ask questions, asking for permission before touching the patient, and stopping the exam at the patient’s request. The video demonstration of giving the patient control over the exam was seen to improve the quality of exams, not only for survivors, but for all patients:

“Now I know, in every stage, when I examine, I ought to tell the patient what I have seen, the result. And I learned that without the patient’s permission, I can’t do anything to him. In fact it was really a new, let’s say a new idea, not only for sexual survivors, but all. I learned things in the video, you know, you never forget.” –Nurse, Kenya

### Treatment and disease prevention

Prior to training, 81% of HCPs correctly identified the 72 hour treatment initiation window for HIV PEP and did not increase following training. The proportion of HCPs who could list the 28 day treatment regimen for HIV PEP increased from 31% to 50% following training (p < .001). The proportion of HCPs who correctly identified the 120 hour treatment initiation window for emergency contraception (EC) increased from 8% to 26% following training (p < .001).

### Caring for male survivors

After training, 80% of HCPs could identify the unique emotional and physical reactions experienced by male survivors, increased from 58% prior to training (p < .001). However, there was no improvement in HCPs’ knowledge of modifications to the physical exam for a male survivor.

### Caring for child survivors

The proportion of HCPs who could identify the unique issues in obtaining informed consent from child survivors increased from 71% to 89% (p < .001) following training and the proportion who could identify the indications for performing a physical exam on a child increased from 41% to 52% (p < .01) following training. 74% correctly identified the age at which girls could be offered emergency contraception, increased from 42% prior to training (p < .001). However, participants’ knowledge of HIV PEP treatment for child survivors did not improve following training.

### Practice

Effects of training on practice were determined by medical record audit conducted before and after the training. A total of 60 cases of sexual assault were retrieved from medical records of health facilities in the three months prior to and following training. Facilities in Ethiopia and Jordan did not have any records of sexual assault cases on file in the three months following training and could therefore not be evaluated. Documented cases were almost exclusively female, and the majority were of reproductive age. Four cases of male survivors were documented in Kenya. Less than half of the cases presented within 72 hours of the assault, the window of time during which they could be eligible to receive both HIV PEP and emergency contraception.

**Figure 1:**
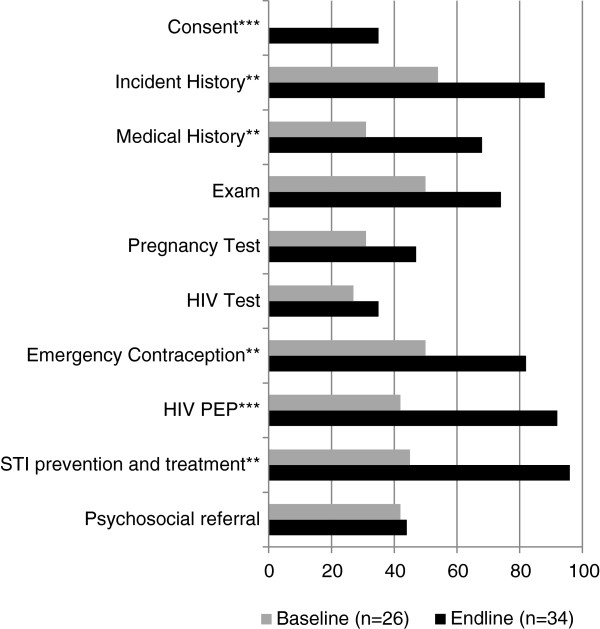
**Proportion of sexual assault survivors treated according to protocol.** **P < .01 ***P < .001. Note: values are proportions of medical records with information documented.

The training seemed to have a positive effect on HCP practice as demonstrated by an increase in eligible survivors receiving emergency contraception from 50% to 82%, HIV post-exposure prophylaxis from 42% to 92%, and STI prophylaxis and treatment from 45% to 96% (Figure 1). In addition to improvements in treatments provided, significant increases were found in patients receiving informed consent, incident history, and medical history. Psychosocial referrals for sexual assault survivors did not improve following training.

## Discussion

This study provided new insight into the gaps in HCPs’ attitudes, knowledge, confidence, and practice in the clinical care for sexual assault survivors in diverse humanitarian settings, suggesting a global need for HCP training on this topic to ensure competent, compassionate, and confidential care is delivered to sexual assault survivors. In addition to demonstrating a need for capacity building in clinical care for sexual assault survivors, the study also demonstrated the effectiveness of the IRC CCSAS training tool in improving HCPs’ attitudes, knowledge, confidence, and practices in diverse humanitarian settings globally. Without training, HCPs lacked positive attitudes, knowledge, and confidence necessary for providing compassionate, competent, and confidential clinical care for sexual assault survivors. These results are consistent with other studies which have shown limited clinical competency and negative attitudes among HCPs inhibit care seeking, lead to poor quality services, and contribute to survivors’ re-traumatization [5-10]. These findings reinforce the acute need for intervention to ensure quality clinical care for sexual assault survivors is provided in keeping with the *Minimum Initial Service Package (MISP) for Reproductive Health in Crisis Situations,* which includes clinical management for survivors of sexual assault and is part of the Sphere Project’s *Minimum Standards in Humanitarian Response*[4]. Training in CCSAS is recommended for all HCPs working in humanitarian settings.

The CCSAS training was effective in improving HCPs’ respect for patient rights but was not effective in changing negative attitudes of blaming and disbelief for persons reporting sexual assault. Although beliefs are hard to change, CCSAS training can improve HCPs’ respect for patient rights. Training did not have a significant effect on HCPs’ beliefs about sexual assault. Negative and potentially harmful attitudes including survivor blame and disbelieving women who report sexual assault continued to be prevalent three months after the intervention. However, the training succeeded at improving the attitudes of HCPs toward their professional role in respecting patient rights, particularly the rights to non-discrimination and self-determination. The human rights framework employed in the CCSAS training has been promoted in other studies as an effective tool for changing the way HCPs treat patients [15]. Findings suggest the CCSAS training may be effective at promoting compassionate clinical care for sexual assault survivors by enhancing HCPs’ respect for patient rights, in spite of persisting negative beliefs. Training will be most effective at promoting compassionate care for sexual assault survivors in the short term by emphasizing respect for patient rights rather than addressing HCP beliefs.

The CCSAS training was effective in improving HCPs’ knowledge and confidence in aspects of direct patient. For example, providers reported that the skills of active listening and giving a patient control over the exam (e.g. encouraging the patient to ask questions and explaining findings during the exam) had improved the quality of history taking and exams for all patients. However, while the majority of providers could identify the active listening skills necessary for conducting a health history, less than one-third could recall questions involved in the history required for directing care (e.g. time of assault, menstrual/obstetric history). These findings underline the importance of a comprehensive medical history and exam form as a job aide to guide the provider in conducting a competent medical history and exam. Additionally, only half of HCPs could list the duration of HIV PEP treatment following training and fewer could recall the treatment initiation window for providing emergency contraception. These findings suggest that written drug treatment protocols are essential to guide the provider in delivery of treatment. Comprehensive national or international protocols must be made easily available in all facilities in the language of the provider and all providers should be made aware of their importance. Despite improvements in HCPs’ practice providing EC, PEP, and STI prophylaxis and treatment, health facility level barriers including stock-outs of HIV PEP at health centers in DRC and policy restrictions on availability of emergency contraception in Jordan remained.

Organizational and contextual factors influenced uptake of the training. The most significant improvements to HCPs’ attitudes were demonstrated in Kenya and Ethiopia, where gender-based violence case managers participated in training with HCPs and provided on-going supportive supervision and follow-up after training. Institutional involvement and commitment has been established as a key element to ensuring facility-wide preparedness to respond to sexual violence. Implications of these findings suggest that the CCSAS training should be conducted in a multi-disciplinary setting and accompanied by on-going supportive supervision and follow-up to ensure improvements in HCP attitudes, knowledge, confidence, and practices. These results are consistent with reviews of other healthcare based interventions which support a systems approach for promoting competent care [7]. Training will be most effective at promoting competent care for sexual assault survivors if coupled with additional facility based interventions including the implementation of proper drug management, treatment protocols, medical history and exam forms, job aides, and supportive supervision. Health facility preparedness is discussed in the second paper of this series: “Clinical care for sexual assault survivors multimedia training: a mixed-methods study of effect on health facility preparedness in humanitarian settings.”

### Study limitations

The main limitations of this study are the quasi-experimental natures of its design as well as its limited sample size. Due to the lack of a control group, we cannot generalize its impact to all providers who may use this multimedia tool. Additionally, because of the short duration of the study, we do not know if the measured changes in knowledge, attitude and confidence levels will be sustained in the longer term. However, because of the diversity of the context and settings of the countries in which it was implemented, we can assume the utility and acceptability of this tool in camp and non-camp settings, and for countries within and outside Africa, thereby increasing the geographical scope of providers who can successfully make use of it.

## Conclusions

This study gives insight into the effectiveness of the IRC CCSAS training tool in four humanitarian settings. The training tool appears useful, as it was positively associated with improvements in HCP respect for patient rights, knowledge, confidence, and clinical practice. The training tool did not appear to be effective at changing negative attitudes among HCPs, including blaming and disbelieving women who report sexual assault. Although beliefs about sexual assault are hard to change, CCSAS training can improve HCPs’ respect for patient rights and knowledge and confidence in direct patient care, contributing to more competent and compassionate clinical care for sexual assault survivors. Findings suggest other agencies working in humanitarian settings could use the CCSAS training tool to build the capacity of HCPs for improved quality clinical care for sexual assault survivors.

## Competing interests

The authors declare that they have no competing interests.

## Authors’ contributions

JRS designed the research protocol and data collection tools, oversaw data collection and analysis, and wrote the first draft of the paper. LSH aided in qualitative data collection and analysis. NM conducted quantitative data analysis. AL designed the training intervention. AS and DP advised and oversaw research study design and analysis. All authors reviewed drafts of the paper, and JRS was responsible for collation of inputs and redrafting. All authors read and approved the final manuscript.
